# St. Augustine Meeting—Abstracts

**Published:** 1898-06

**Authors:** J. M. Walker


### St. Augustine Meeting—Abstracts.

Reported for the DENTAL REGISTER by Mrs. J. M. Walker.

INCIDENTS OF OFFICE PRACTICE.

In the discussion of minor accidents and the necessity of antiseptic precautions, DR. J. Y. CRAWFORD said that whenever he punctured a finger with an instrument, in use in the mouth, he immediately wrapped it closely with a ribbon of bibulous wrapping, that with floss silk, and then immersed the whole in alcohol, and in no case did he have any ill results from wounds caused by instruments. He does not attribute this to the anesthetic effect of the alcohol, his idea being that when the alcohol is absorbed into the system *pari passe* with the toxin, there is elaborated an anti-toxin within the organism—something which annihilates the toxic influence.

DR. WALKER related a successful case of transplantation. Two second bicuspids which had been extracted three weeks previous, in a regulating case being placed in the sockets of the two right superior bicuspids, the patient having presented with the roots of these teeth incurably abrased and the crowns gone. The implanted teeth were retained by a bandage made before the roots were extracted, according to Dr. Jack's method. No saliva was allowed to enter the sockets, which were not syringed, the teeth being implanted while the blood was still flowing. There was no perceptible swelling and the case did well from the start, assuming a perfectly natural appearance within a few days. After a lapse of two years there is every appearance of permanent success.

DR. B. HOLLY SMITH described a case in which two months after the crown of a tooth had been accidentally broken, the pulp still vital and the patient objecting to its removal, the root was banded and an English porcelain face with gold backing adapted, with very satisfactory results.

DR. T. P. HINMAN described a case in which, with a perfect crown with enamel intact, there was a spot at the cervical margin of the labial surface, extending under the gum, which showed red through apparently thin enamel, and which, when opened into, revealed a large cavity filled with an apparent enlargement of the pulp, which bled profusely when penetrated.

DR. CRAWFORD described a left central incisor which had been broken diagonally across, by a kick from a mule, and the fragments of the tooth had grown together again. Dr. Crawford also described another very peculiar case. Spontaneous abrasion had caused a shallow cavity about the treatment of which he was undecided; whether to cut out sufficient to fill with gold, or to insert a porcelain inlay. While the question was in abeyance the patient visited Philadelphia, when Dr. C. N. Peirce saw the tooth and pronounced it "a dead tooth." On the return of the patient Dr. Crawford was informed of Dr. Peirce's judgment, which, however, was not confirmed in Dr. Crawford's opinion. He decided to fill the cavity, and on cutting into it found no sensation. Cutting to the pulp cavity it was found to be absolutely vacant, but at the point of junction of crown and root there was found a disk of bone as perfect as if it had been artificially inserted—a perfectly established partition. Beyond this there was still no sensation and no signs of pulp tissue. The tooth was treated antiseptically and filled with cement, with a porelain inlay at the labial surface. It has never given any trouble since.

